# Pars plana vitrectomy combined with penetrating keratoplasty and transscleral-sutured intraocular lens implantation in complex eyes: a case series

**DOI:** 10.1186/s12886-020-01639-y

**Published:** 2020-09-14

**Authors:** Takahiko Hayashi, Ida Yasutsugu, Toshiki Shimizu, Tsubasa Kuroki, Yuji Kobashigawa, Yasuhito Iijima, Kentaro Yuda

**Affiliations:** 1 Department of Ophthalmology, Yokohama Minami Kyosai Hospital, 1-21-1, Mutsuura Higashi, Yokohama, Kanagawa 236-0037 Japan; 2 Department of Ophthalmology, University of Cologne, Cologne, Germany; 3 Department of Ophthalmology, Jichi Medical University, Tochigi, Shimotsuke Japan; 4 Department of Ophthalmology, Yokohama City University School of Medicine, Yokohama, Kanagawa Japan; 5 Department of Technology and Design Thinking for Medicine (DT2M), Hiroshima University, Hiroshima, Japan; 6 Yokosuka Chuo Eye Clinic, Yokohama, Kanagawa Japan; 7 Sofukai Oppama Ekimae Eye Clinic, Yokohama, Kanagawa Japan; 8 Angiogenesis Laboratory, Department of Ophthalmology, Massachusetts Eye and Ear Infirmary, Harvard Medical School, Boston, USA; 9 Kikuna Yuda Eye Clinic, Yokohama, Kanagawa Japan

**Keywords:** Penetrating keratoplasty, Pars plana vitrectomy, Intraocular lens implantation, Transscleral-sutured intraocular lens

## Abstract

**Background:**

The aim of this study was to evaluate the clinical outcomes of pars plana vitrectomy (PPV) combined with penetrating keratoplasty (PKP) and transscleral-sutured intraocular lens (IOL) implantation (IOL-suture) in complex eyes.

**Methods:**

In this prospective, consecutive interventional case series, patients who underwent PKP combined with PPV and IOL implantation from July 2014 to March 2018 at Yokohama Minami Kyosai Hospital were enrolled. The postoperative best corrected visual acuity (BCVA) (converted to logarithm of the minimal angle of resolution [logMAR] units), intraocular pressure (IOP, mmHg), endothelial cell density (ECD, cells/mm^2^), graft survival, complications, astigmatism, and spherical equivalent (dioptres [D]) were evaluated.

**Results:**

This study included 11 eyes of 11 patients (three females and eight males; mean age, 61.8 ± 13.9 years) with an injury (*n* = 6) or bullous keratopathy (*n* = 5). The BCVA significantly improved from 1.50 ± 0.66 logMAR preoperatively to 0.78 ± 0.59 logMAR (*p* < 0.001) postoperatively. The baseline ECD significantly decreased from 2396 ± 238 cells/mm^2^ preoperatively to 1132 ± 323 cells/mm^2^ (*p* < 0.001) postoperatively. Despite two rejection episodes, graft survival rates were 100%. The mean follow-up period was 38.0 ± 20.5 months. Two patients required combined glaucoma surgery, and three patients underwent subsequent glaucoma surgery. Postoperative astigmatism and spherical equivalent were 3.9 ± 3.2 D and 0.29 ± 2.18 D, respectively.

**Conclusion:**

The combination of PKP, PPV, and IOL-suture implantation could be a safe and effective approach for eyes requiring anterior segment surgery; however, these eyes are associated with a higher incidence of glaucoma surgery.

## Background

Corneal transplantation has been the most common type of organ transplantation over the last century [1, 2]. Despite the increasing number of lamellar surgeries such as deep anterior lamellar keratoplasty (DALK), endothelial keratoplasty (EK), Descemet’s stripping automated endothelial keratoplasty (DSAEK), and Descemet’s membrane endothelial keratoplasty, approximately 40% of all keratoplasties performed are penetrating keratoplasties (PKPs) [3, 4].

There are downsides to PKP, such as suture-related problems (higher astigmatism or infection), transplant rejection, glaucoma (steroid-dependent), and rupture due to injury [3]. Despite these problems, [5–7] PKP could drastically improve the sight of patients with severe corneal disease or damage [8].

Simple stromal opacity and corneal oedema caused by endothelial dysfunction could be treated with DALK and EK, respectively [9, 10]. However, most cases that require PKP are complex, and patients can have a history of corneal injury or infection. These cases may require multiple procedures such as a vitrectomy for vitreous problems including vitreous prolapse, vitreous haemorrhage, or retinal detachment; iris reconstruction for an iris defect or angle closure; intraocular lens (IOL) implantation for aphakia; or glaucoma surgery for progressive glaucoma [8, 11–16]. In these situations, simultaneous surgeries could be beneficial to the patient. For example, a full vitrectomy could prevent retinal detachment, and iris reconstruction and IOL implantation could be performed for visual recovery. In cases of extremely high intraocular pressure (IOP) that are resistant to drug therapy, a combined glaucoma surgery might be essential to prevent the progression of glaucoma [17–22].

Herein, this case series investigates the surgical technique and clinical course of PKP combined with pars plana vitrectomy (PPV) and transscleral-sutured intraocular lens implantation (IOL-suture). To the best of our knowledge, this is the first study to investigate this approach and report the good outcomes of simultaneous complex ocular surgeries.

## Methods

### Study design

This prospective study was approved by the institutional Review Board (approval no. YKH_26_05_12) and adhered to the tenets of the Declaration of Helsinki. The study procedures followed all institutional guidelines, and all patients provided informed consent. Patients requiring PKP combined with PPV and IOL-sutures from July 2014 to March 2017 were enrolled.

### Surgical technique

**Fig. 1:**
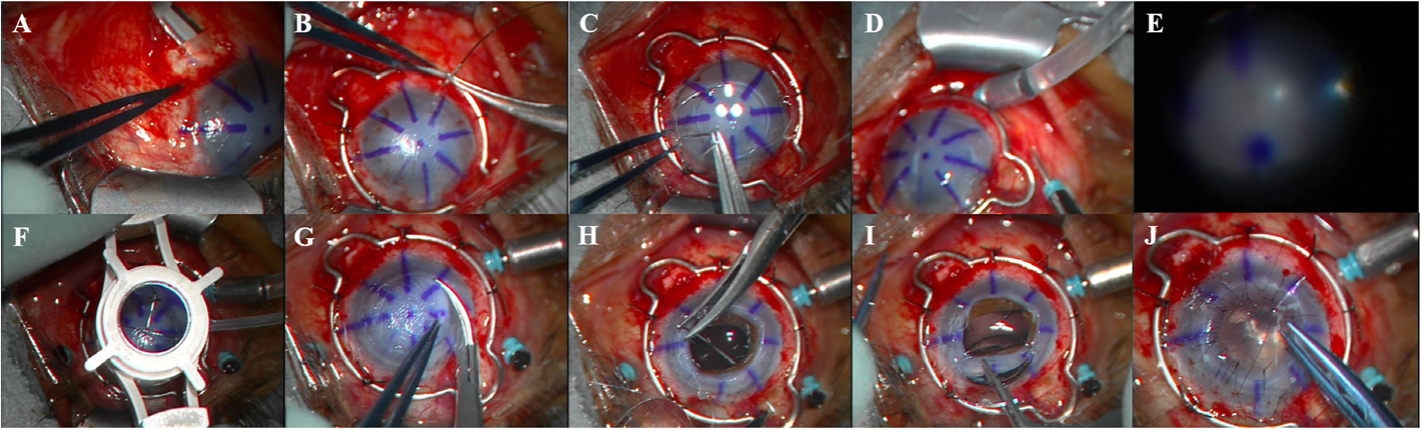
Design of combined penetrating keratoplasty (PKP), pars plana vitrectomy (PPV), and intraocular lens (IOL)-suture surgery **a** The surgical design should be determined prior to surgery including where to place the scleral ring and whether to create two scleral flaps or three-ports. After determination of the surgical design, two scleral pockets for the intraocular lens (IOL) suturing are created at the surgeons’ preference. **b** A scleral ring (Nishida ring, Inami) is sutured to the sclera with 6–0 silk. **c** Two 10–0 polypropylene sutures (PC-9) are fixated to the top of the IOL loop (CZ, Alcon) before the next procedure. **d** Three-ports are created using a 25-gauge trocar (Alcon), and the infusion canula was set. **e** Using the wide-viewing system, core vitrectomy and peripheral vitrectomy is performed. **f**, **g** Using a trephine and Katzin Scissors, the host cornea is removed. **h** In the open-sky technique (partial dissection), the IOL is sutured to the two scleral flaps 1.5 mm from the limbus. **i** The IOL is placed at the back of the iris and is centred. **j** The donor graft is sutured using 10–0 nylon

All surgeries were performed under general anaesthesia. All of the surgical steps are shown in Fig. 1. The surgical design was determined prior to the surgical steps. Firstly, two scleral tunnels were created after resecting the conjunctiva. A scleral ring for combined surgery (Nishida scleral ring, Inami, Tokyo, Japan) was sutured to the sclera with 6–0 silk (Mani, Tochigi, Japan), and 10–0 polypropylene sutures were fixed to the loop of the IOL (CZ70BD®, Alcon, Fort Worth, TX) on both sides using the cow-hitch technique [23]. PPV was performed using a 25-gauge (Constellation; Alcon, Fort Worth, TX) and a wide-viewing system (Resight 500; Carl Zeiss Meditec, Jena, Germany) in closed system (not open-sky) prior to trephination by retina specialists. Even in patients with a history of vitrectomy, the residual vitreous was checked and removed completely by shaving the vitreous base. We performed a complete PPV, stopped the infusion, and reduced the IOP prior to trephination to prevent vitreous prolapse or suprachoroidal haemorrhage. The host cornea was cut using a trephine (Katena, Denville, USA) at 7.5 mm. The donor graft was prepared using a donor punch (Katena, Denville, USA) at either 7.75 mm or 8.0 mm. Using the open-sky PPV technique, the 10–0 polypropylene needles pierced the scleral flap 2.0 mm from the limbus. The donor graft was sutured using 10–0 nylon (Mani, Tochigi, Japan). The appropriate amount of cohesive ophthalmic viscosurgical devices (OPELEAD® HV [0.85] 1%) was used during the procedure.

### Postoperative care

Postoperative medications included 1.5% levofloxacin (Cravit) for 2 weeks, 0.1% betamethasone sodium phosphate (Sanbetasone; Santen) for 3 months, and 2% rebamipide ophthalmic solution (Mucosta; Otsuka, Japan, Tokyo) for 3 months, starting at 4 times per day and tapered thereafter. If necessary, glaucoma agents were applied.

### Patients and examinations

Besides the standard examination using slit-lamp microscopy, the following main outcomes were evaluated both preoperatively and postoperatively in all eyes: best spectacle-corrected visual acuity (BCVA) converted to logarithm of the minimal angle of resolution (logMAR) units, intraocular pressure (IOP, mmHg), and endothelial cell density (ECD, cells/mm^2^). Graft survival, complications, astigmatism, and the spherical equivalent were also evaluated (measured in dioptres [D]). The main outcome results for all the included cases are shown in Table 1.

**Table 1:** Patient Characteristics

Case	OD/OS	Aetiology	Type of surgery	Previous surgeries	Additional surgery	Pre VA	Post VA
1	OD	Bullous keratopathy	PKP + re-PPV +IOL-suture+GDD	Trabeculectomy (failed), PEA+PPV + Silicon oil	No	CF	20/2000
2	OD	Ocular trauma	PKP + re-PPV +IOL-suture	PEA+PPV	GDD	20/29	20/23
3	OS	Ocular trauma (perforation)	PKP + re-PPV +IOL-suture	PEA+PPV + Corneal suture	No	20/400	20/250
4	OS	Ocular trauma (perforation)	PKP + PPV +IOL-suture	PEA+Corneal suture	No	20/2000	20/220
5	OS	Bullous keratopathy	PKP + re-PPV +IOL-suture+GDD	PEA+PPV + Silicon oil, Silicon oil removal	No	20/2000	20/250
6	OS	Bullous keratopathy	PKP + PPV +IOL-suture	ECCE, PKP (twice)	No	20/100	20/29
7	OS	Ocular trauma	PKP + PPV +IOL-suture	PKP	Trabeclectomy	20/400	20/100
8	OS	Bullous keratopathy	PKP + re-PPV +IOL-suture	PEA+PPV + Corneal suture, PKP	GDD	20/2000	20/50
9	OD	Bullous keratopathy	PKP + PPV +IOL-suture	PKP + ECCE	No	20/600	20/130
10	OS	Ocular trauma	PKP + PPV +IOL-suture	PEA+PPV +Silicon oil injection +Corneal suture, Silicon oil removal	No	20/2000	20/50
11	OS	Ocular trauma	PKP + PPV +IOL-suture	PEA+Corneal suture	No	20/600	20/130

All patients underwent pars plana vitrectomy with penetrating keratoplasty and transscleral-sutured intraocular lens implantation

*OD* right eye, *OS* left eye, *Pre* preoperative, *VA* best corrected visual acuity, *Post* postoperative, *PKP* penetrating keratoplasty, *PPV* pars plana vitrectomy, *IOL-suture* transscleral-sutured posterior chamber intraocular lens implantation, *GDD* glaucoma drainage device, *PEA* phacoemulsification and aspiration, *ECCE* extracapsular cataract extraction, *CF* counting fingers

### Statistical analyses

Statistical analyses were performed using JMP Pro software version 14.0.0 (SAS Institute, Cary, NC, USA). Statistical significance was defined as *p* < 0.05. All average values are described as mean ± standard deviation. For the statistical analyses, BCVA was converted to logMAR units. Regarding poor visual acuity, the logMAR values were translated to light perception, logMAR = 2.8; perception of hand motions, logMAR = 2.3; and counting fingers, logMAR = 2.0 [24]. The Mann-Whitney U test was used to compare the preoperative and postoperative outcomes (BCVA, IOP, astigmatism).

## Results

### Patient characteristics

Three female and eight male patients, with an average age of 61.8 ± 13.9 years, took part in this study. The corneal aetiology was either injury (*n* = 6) or bullous keratopathy (*n* = 5). The average follow-up period was 38.0 ± 20.5 months (Table 1).

### Clinical course (visual recovery and endothelial cell density)

The BCVA (converted to logMAR units) significantly improved from 1.50 ± 0.66 logMAR preoperatively to 0.78 ± 0.59 logMAR (*p* < 0.001) postoperatively. The baseline ECD (ECD of the donor tissue) significantly decreased from 2396 ± 238 cells/mm^2^ preoperatively, to 1132 ± 323 cells/mm^2^ (p < 0.001) postoperatively. Figure 1 shows the graft survival for this case series. Postoperative astigmatism and spherical equivalent were 3.9 ± 3.2 D and 0.29 ± 2.18 D, respectively.

### Complications

The baseline IOP was 15.8 ± 12.9 mmHg preoperatively. Five cases required glaucoma surgery either simultaneously or postoperatively. Two patients had an IOP over 40 mmHg and required combined glaucoma surgery. Three patients had an increased IOP that was resistant to anti-glaucomatous agents and underwent subsequent glaucoma surgery (two patients had pars plana glaucoma drainage devices implanted, and one had filtrating surgery). The postoperative IOP was stable (12.7 ± 3.6 mmHg). One patient developed an epiretinal membrane and required membrane removal. Two eyes showed reversible graft rejection, and there was no graft failure.

## Discussion

The current study shows our technique for a pars plana approach, such as PPV, combined with PKP. All procedures were performed without complications. Under a wide-viewing system, PPV could be completed without the use of an artificial cornea. Any residual vitreous base following PPV could be removed with the shaving technique. Without treatment these patients with complex needs may have gone blind, but following the combined surgery, the visual function of the patients significantly improved, and the mid-term survival rates were excellent. There were no cases of retinal detachment following surgery. Despite the improvement in corneal transparency and visual recovery, the requirement for additional glaucoma surgeries was relatively high. Patients may have a better quality of life if IOL implantation and PKP are performed simultaneously.

There are three suggestions regarding the importance and the efficacy of the posterior approach during PKP: First, the prevention of retinal detachment (including proliferative vitreous retinopathy) is very important. It seems appropriate that retina specialists perform PPV in combination with PKP to avoid the risk of retinal detachment following an incomplete vitrectomy such as an anterior vitrectomy in the posterior capsule rupture, or IOL-suture. If there is a complication in the posterior segment during PPV, such as a retinal break, it can be rapidly addressed [25].

Second, the posterior approach in simple cases could be performed without difficulty. Since our case series did not include severe corneal opacity, all procedures could be performed with a wide-viewing system (without any artificial corneas such as Eckardt temporary keratoprosthesis) in closed system prior to trephination (not open-sky vitrectomy). Performing the vitrectomy via closed system PPV rather than open-sky vitrectomy allowed us to reduce the time of open-sky status, an advantage of our procedure. The use of PPV with a temporary keratoprosthesis has been reported. Yokogawa et al. published a report on the combined treatment of PKP or DSAEK using an artificial cornea [8, 15, 16]. In our experience, we had no trouble performing a simple PPV, including the creation of a posterior vitreous detachment and shaving of the peripheral vitreous body.

Third, the incidence of globe collapse could be reduced by the use of posterior infusion. Using this approach, the globe could be well-maintained without sudden collapses.

The results of this study show that the combined procedure of PKP, PPV, and IOL-suture could be a safe and effective approach for patients requiring anterior segment surgery. Despite the increasing number of scleral fixations, [26] in the present study the preferred method was an IOL-suture.

There were limitations to this study, such as the relatively small number of participants, differing aetiologies, and different treatment protocols. First, the lack of long-term follow-up is the most important limitation. Despite suture breakage after IOL suture being one of the major late complications (especially when using 10–0 polypropylene), [27] our follow-up period was relatively short. Second, our procedure was performed with the help of retina specialists. Although the PPV portion was performed by retina specialists with very good results, it is impossible to compare our method to anterior vitrectomy alone or PPV performed by anterior segment surgeons. Thirdly, postoperative glaucoma developed in 5 cases requiring glaucoma surgery. Two cases required an Ahmed valve, one needed a Baerveldt implant, and one case needed filtrating surgery (trabeculectomy) after PKP. One case was treated with a simultaneous glaucoma implant, (Baerveldt) PKP, PPV, and IOL-suture surgery. According to past reports, the combined surgery of PKP and Ahmed valve implantation had a negative impact on graft survival. However, in the present study, the glaucoma shunt tubes were placed into the pars plana, because tube implantation to the pars plana has been shown to result in better corneal graft survival rates and reduce complications compared with implantation into the anterior chamber [28–32]. Since the type of glaucoma surgery was selected according to either the patient’s condition or the glaucoma surgeon’s preference, only one case was treated by trabeculectomy. In the future, long-term studies regarding the correlation between glaucoma surgery and keratoplasty will be necessary for further development.

## Conclusion

In conclusion, the current study advocates the importance and efficacy of the posterior approach combined with PKP for anterior segment surgeons.

## Data Availability

The datasets used and/or analysed during the current study available from the corresponding author on reasonable request.

## Abbreviations

PPV: Pars plana vitrectomy
PKP: Penetrating keratoplasty
IOL: Intraocular lens
IOL-suture: Transscleral-sutured intraocular lens implantation
DALK: Deep anterior lamellar keratoplasty
EK: Endothelial keratoplasty
DSAEK: Descemet’s stripping automated endothelial keratoplasty
BCVA: Best corrected visual acuity
logMAR: Logarithm of the minimum angle of resolution
ECD: Endothelial cell density
IOP: Intraocular pressure
